# Pseudo-allergic reactions induced by Chinese medicine injections: a review

**DOI:** 10.1186/s13020-023-00855-0

**Published:** 2023-11-13

**Authors:** Fanmei Zou, Qiuzheng Du, Yuanyuan Zhang, Lihua Zuo, Zhi Sun

**Affiliations:** 1 Department of Pharmacy, The First Affiliated Hospital of Zhengzhou University, Zhengzhou, 450052 Henan Zhengzhou China; 2 Henan Engineering Research Center of Clinical Mass Spectrometry for Precision Medicine, Zhengzhou, 450052 Henan Zhengzhou China

**Keywords:** Traditional Chinese medicine injections, Pseudo-allergic reactions, Mechanism, Material basis, Allergenic ingredients

## Abstract

Traditional Chinese medicine injections (TCMIs) is a new dosage form of Chinese medicine, which plays a unique role in rescuing patients with critical illnesses that are difficult to replace. With the rapid development and widespread application of TCMIs in recent years, their adverse events have emerged and attracted much attention. Among them, pseudo-allergic reactions, i.e., the most significant adverse reactions occurring with the first dose without immunoglobulin E mediated conditions. Currently, studies on the types of TCMIs and antibiotic mechanisms that cause pseudo-allergic reactions are incomplete, and standard models and technical guidelines for assessing TCMIs have not been established. First, this review describes the causes of pseudo-allergic reactions, in which the components and structures responsible for pseudo-allergic reactions are summarized. Second, the mechanisms by which pseudo-allergic reactions are discussed, including direct stimulation of mast cells and complement activation. Then, research models of pseudo-allergic reaction diseases are reviewed, including animal models and cellular models. Finally, the outlook and future challenges for the development of pseudo-allergic reactions in traditional Chinese medicine (TCM) are outlined. This shed new light on the assessment and risk prevention of pseudo-allergic reactions in TCM and the prevention of clinical adverse reactions in TCM.

## Introduction

Currently, allergic diseases are a global public health problem. Allergic diseases are commonly considered to be immunoglobulin E (IgE)-mediated hypersensitivity reactions, but in recent years, studies have found that pseudo-allergic reactions are also an important part of allergic reaction [1]. Both type I and pseudo-allergic reactions are caused by the release of reactive mediators from activated mast cells or basophils. However, anaphylactoid reactions, also known as pseudo-allergic reactions, or non-allergic drug hypersensitivity, is a non-IgE mediated allergic reaction when drugs and additional antigens first stimulate the body, it can directly produce anaphylaxis symptoms [2]. At present, drugs that can cause anaphylaxis mainly include the following basic drugs. Such as traditional Chinese medicine injections [3], contrast agents, liposome nanocapsules, non-steroidal anti-inflammatory drugs [4], monoclonal antibodies, neuromuscular blockers, anesthetics, antibiotics, etc. TCMIs is a novel dosage form developed based on Chinese medicine preparations, which has a rapid onset of effect and favorable efficacy [5]. Conversely, more and more adverse drug reaction (ADR) events may even seriously harm the lives of patients [6]. The main adverse reactions of TCMIs were allergic reactions, of which about 77% were pseudo-allergic reactions [2].

Since 1965, Liu Gengtao et al. first studied pseudo-anaphylactic reactions to TCMIs [7]. Subsequently, there was a large number of literature studies, and at present, TCMIs such as Shuanghuanglian injection, Qingkailing injection, Houttuynia injection, Chuardauning injection and Xuebijing injection have been investigated, and thus the field has attracted the attention of researchers [8].

So far, numerous instances of pseudo-allergic reactions of TCMIs have been reported in different fields, including anesthesia [9, 10], hemostasis [11], etc. Although pseudo-allergic reactions of TCMIs have been extensively studied and summarized in some excellent reviews [12, 13]. Yang et al. [14] presented a review focusing on the mechanism aspects of pseudo-allergic reactions of TCMIs. However, it only covered some aspects of pseudo-allergic reactions of TCMIs. In addition, Li Hancheng’s team summarized animal models of pseudo-allergic reactions induced by TCMIs [12]. Therefore, we focus on reports related to the mechanisms and experimental models of pseudo-allergic reactions induced by TCMIs.

**Fig. 1:**
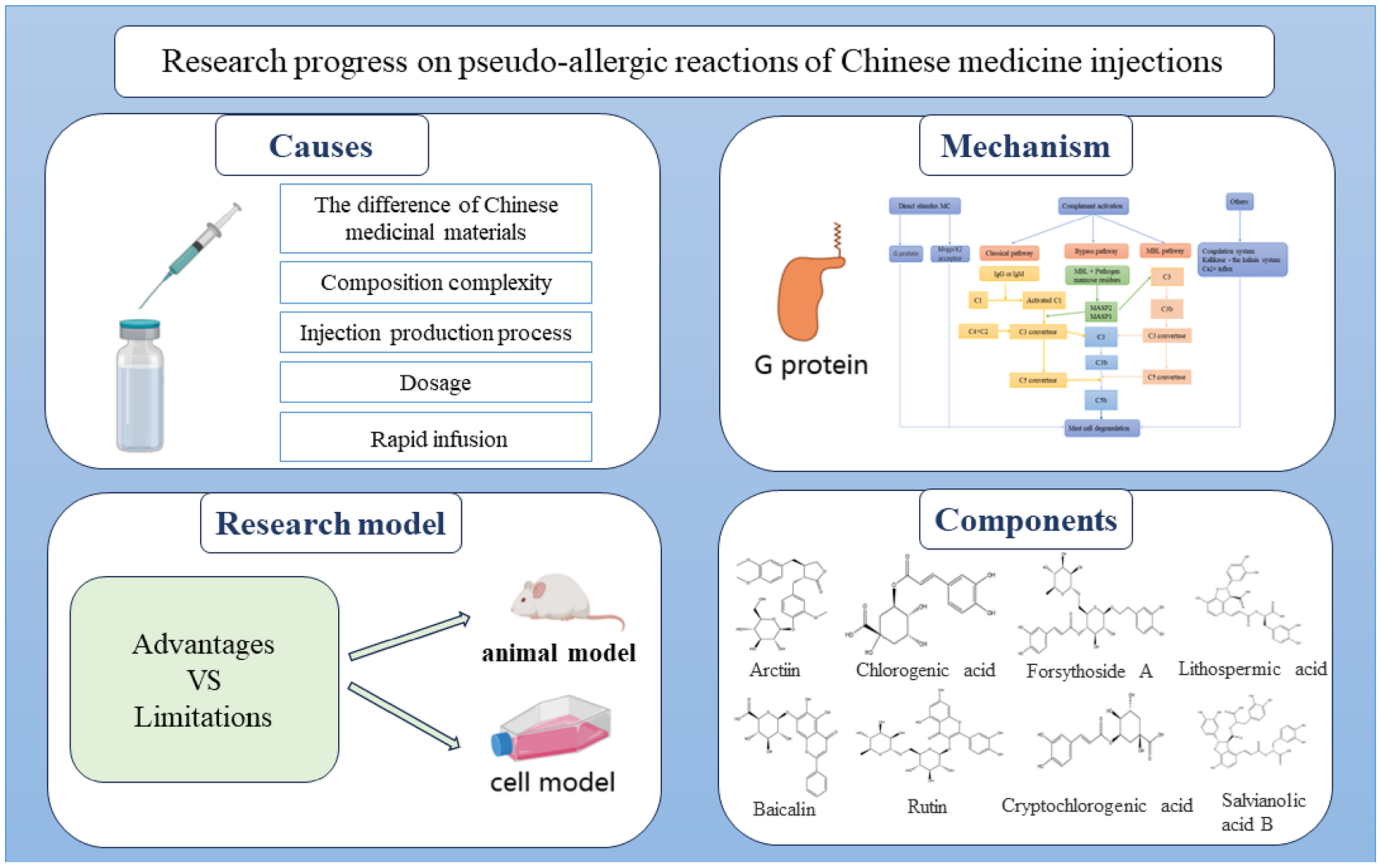
The main elements of this review

Here, we intend to summarize the work related to the induction of pseudo-allergic reactions by TCMIs comprehensively, covering the content of 1965 to the present, and discuss the causes and mechanisms of action of pseudo-allergic reactions induced by traditional Chinese medicine injection from different angles, and then the classification and application of specific experimental models. We first summarize the mechanisms of action of TCMIs-induced pseudo-allergic reactions, and then compare the properties of animal and cell models. Then, the allergenic components are reviewed based on previous studies. Finally, the conclusions drawn in this review are summarized, and the main challenges that may be faced in the future for pseudo-allergic reactions induced by TCMIs are outlined. The main elements of this work are illustrated in Fig. 1.

## Causes of anaphylactic reactions

### The difference of Chinese medicinal materials

The important reasons for the occurrence of pseudo-allergic reactions in TCMIs include differences in the original medicinal materials [15]. Firstly, the distinctive origins of medicinal materials can result in variations in the content of active ingredients, organic acid content, aromatic substances, volatile oils, pigments, and other components. Secondly, factors like harvesting season, soil quality, and climate can also contribute to differences in the ingredients contained in medicinal materials, such as the content of active ingredients and pigments. As a result, the quality of the same drug from different sources may be different. The unstable quality of raw medicinal materials of TCMIs commonly leads to the quality difference between different batches, which can lead to different adverse reactions such as allergic and pseudo-allergic reactions.

### Composition complexity

The composition of TCM is quite complex and includes some components with large molecular weight, such as certain macromolecular substances, animal and plant proteins, etc., which are commonly used as allergens and can cause allergies and allergic reactions. For example, the buffalo horn component in Qingkailing injection is considered to be an allergen [16]. Moreover, when medical personnel use traditional Chinese medicine injections, if there is no unreasonable compatibility, insoluble particles in the injections will be increased, thus leading to the occurrence of adverse reactions such as allergies [5]. At present, domestic and foreign studies have shown that Shuanghuanglian injection, Qingkailing injection, Shengmai injection, Sodium Aescinate for injection, Ligustrazine Phosphate injection, Xuesaitong injection and other TCMIs are easy to cause allergic reactions, among which chlorogenic acid, tween-80, tannic acid, > 10 kDa molecule (protein), baicalin and other components can cause allergic reactions [17, 18]. The allergenic components and structures in TCMIs are summarized in Table 1.

**Table 1:** Chinese medicine injection categories, allergic ingredients and structure

TCMI	Allergic ingredients	Structure	Classify	References
Shuanghuanglian injection	Arctiin		Lignin lactones	Yi Y, [19]
	Forsythoside A		Phenylethanoid glycosides	
	Chlorogenic acid		Organic acid	Wang F, [20]
	Cryptochlorogenic acid		Organic acid	
	Baicalin		Flavonoids	Zhang Q, [21]
	Rutin		Flavonoids	
Qingkailing injection	Panax quinquefolium extract and Gardenia jasminoides extract	\	\	Yi Y, [22]
Xiangdan injection	Salvianolic acid B		Phenols	Pang F, [23]
	Lithospermic acid		Polyphenols	
Shuxuening injection	Ginkgo biloba extract	\	\	Yi Y, [24]
Houttuynia injection	Polysorbate 80	\	\	Bao Min, [25]
Xue-Sai-Tong injection	Proteins with over 10 KDa of molecular weight	\	\	Xiang Z, [26]

### Injection production process

The composition of injections can vary due to different manufacturers, processing processes, and preparation technologies. This variation can result in different levels of protein, resin, tannin, and other impurities in the preparation. To improve solubility and stability, additives such as cosolvents and stabilizers are commonly used during production. However, these additives can sometimes cause allergic reactions. For example, the addition of tween-80, which enhances drug solubility, has been found to cause allergic reactions [27]. Additionally, the drug itself can undergo decomposition, oxidation, polymerization, reduction, and other reactions, leading to the formation of impurities that can act as allergens and trigger allergic reactions in the body.

### Dosage

Traditional Chinese medicine is widely believed to be derived from natural sources and is considered to have mild medicinal properties with minimal toxic side effects. However, this belief has led to a common practice among doctors where high doses of medicine are administered without fully understanding the condition of the patients or adjusting the dosage according to individual differences. Consequently, the risk of adverse reactions due to the large dosage of medicine is significantly increased. Moreover, some doctors habitually use medical terms such as “po” and “tid” in prescriptions, which can lead to misunderstandings regarding the appropriate usage and dosage, particularly among patients who are unfamiliar with these terms [28, 29]. In a study conducted by Li et al., the correlation between dosage and pseudo-allergic reactions was investigated for Shuanghuanglian injection and Shenmai injection. The findings indicated that as the dose increased, the severity of pseudo-allergic reactions in mice also increased [30, 31].

### Combination of medication and rapid infusion

Due to the complex composition of TCMIs, chemical composition reactions and pH value changes may occur in the combination of TCMIs, increasing the insoluble particles. When these particles enter blood vessels, they can cause a series of adverse reactions to Chinese herbal injections. In addition, simultaneous and rapid infusion of multiple drugs can make the vascular wall in a state of high-pressure stimulation, aggravating the damage to the vascular wall and causing adverse reactions [32]. Therefore, TCMIs should not be compatible with other medications and should be used separately whenever possible. If compatibility is required, it should also be used after a certain period, to prevent the mixing of several drugs in the blood and lead to adverse reactions [33].

### Other factors

Additional factors contributing to adverse reactions in TCMIs include prolonged storage time after preparation, organism factor and improper solvent, among others. The greatest safety hazard of intravenous infusion of TCMIs is excessive insoluble particles. In the compatibility of intravenous injection and infusion, due to the complex composition and different preparation technology of Chinese herbal medicine, some components exist in the colloidal form in the liquid medicine, and after the compatibility of medicine and infusion, oxidation and polymerization occur and particles are precipitated, or the pH of some Chinese herbal injection is acidic. A large number of insoluble particles may be produced due to salt-out after mixing with 0.9% sodium chloride injection. Similarly, some Chinese herbal injections are alkaline, so they should be avoided when mixed with liquids with excessively low a pH to avoid the precipitation of the effective ingredient. In addition to increased production of insoluble particles, ions in the solvent may affect infusion stability due to salting out or complexation. Studies have shown that with the extension of storage time after preparation, the allergic reactions of mice induced by Shuanghuanglian for injection tend to worsen [34]. In addition, the contents of most drug instructions are incomplete and the warning strength needs to be strengthened [35].

Also, due to the lack of safety studies on TCMIs, such as allergoid and allergic reactions caused by them, and cardiotoxicity, studies have not been conducted in sufficient depth.

## Study on the mechanism of pseudo-allergic reactions

**Fig. 2:**
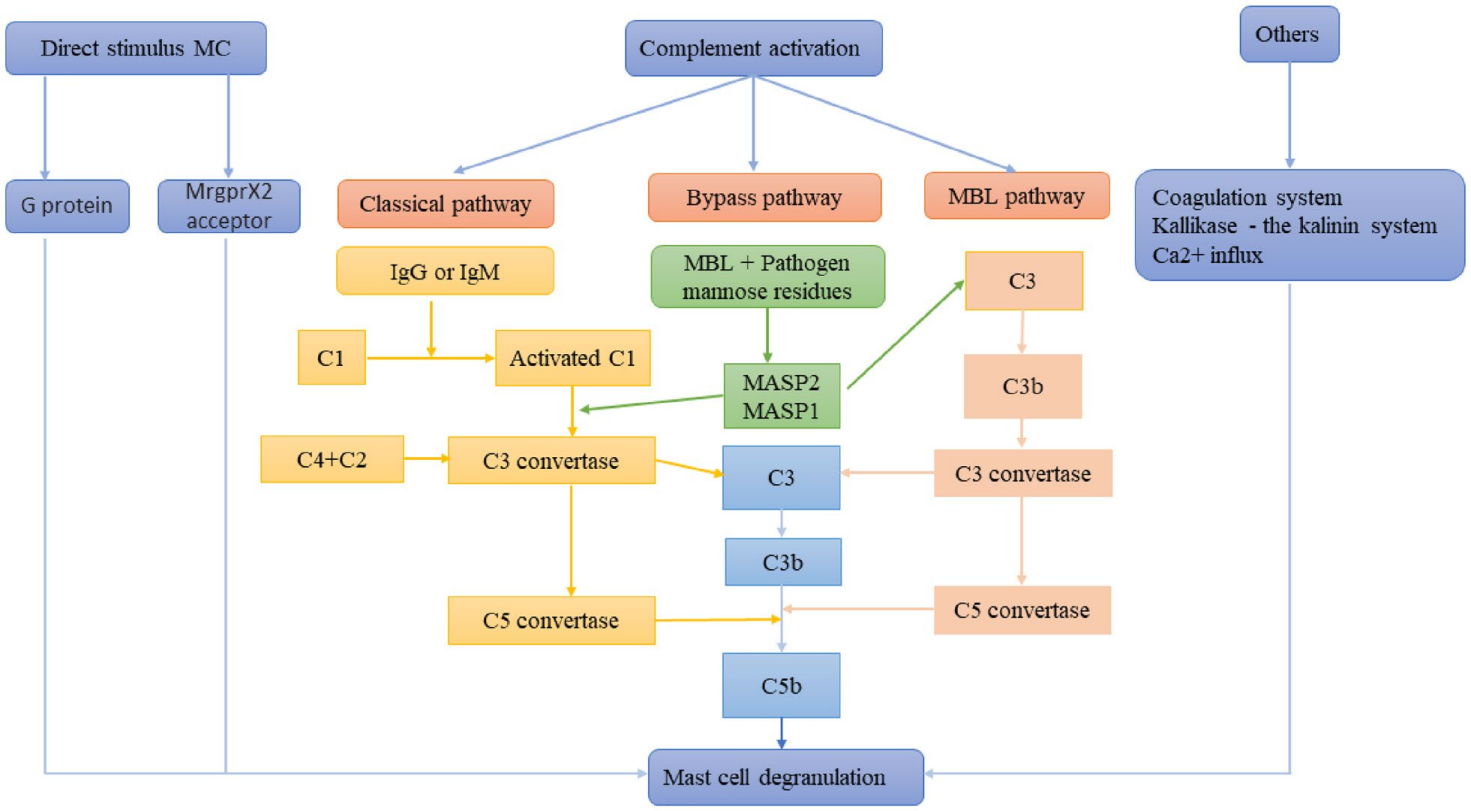
Mechanism of pseudo-allergic reactions in Chinese medicine injection

There are currently several pathways involved in the mechanism of pseudo-allergic reactions. Domestic and foreign studies have found that the mechanism of pseudo-allergic reactions in TCMIs include direct stimulation of mast cells, complement activation, coagulation system and kininogen kinin system, among others. The mechanism of the TCMI induced pseudo-allergic reaction is summarized in Fig. 2.

### Direct stimulus mast cell

Mast cells (MCs) are highly granulated tissue dwelling cells, widely distributed throughout the body in connective tissues and on mucosal surfaces. The direct stimulation of MC was found to be associated with the Mrgprb2 receptor and G protein. Sun et al. [36] used Mrgprb2 knockout mouse model, using histamine, β-Hex release, mast cell degranulation and hind paw swelling test as observation indexes, and found that Mrgprb2 played an important role in Houttuynia cordata injection-induced pseudo-allergic reactions. Subsequently, the Wei D group found that the presence of the human MAS-associated G-protein-coupled receptor X2 (MrgprX2) on the surface of mast cells can mediate degranulation, which also increases the feasibility of studies related to pseudo-allergic reactions [37].

MCs play a crucial role during all the phases of allergic inflammation, releasing histamine and other mediators [38]. Histamine is an amine formed by decarboxylation of histidine dehydrogenase in MCs and eosinophilic granulocyte golgi, playing an crucial role in the inflammatory response [39]. Heparin and chondroitin sulfate combine with histamine, protease, and acid hydrolase through sulfate group to form the main supporting matrix of mast cell granules [40]. Upon mast cell activation, histamine is released extracellularly by exchanging sodium ions with the extracellular environment, thereby exerting its biological functions such as bronchial smooth muscle constriction, increased mucus secretion, and increased vascular permeability [41].

β-Hexosaminidase (β-Hex) is one of more than 40 enzymes present in lysosomes that are involved in the degradation of glycoproteins, glycolipids, and aminoglyaccharides by hydrolyzing β-linked acetylglucosamine and N-acetylgalactosamine residues [42]. At the same time, β-Hex is also an active substance stored in mast cell secretory granules. Studies have shown that when mast cells are activated and degranulated, β-Hex is released parallel to histamine, thus this index is also commonly used as a marker of mast cell activation and degranulation in foreign studies [43]. Due to the sensitivity, accuracy, convenience and reproducibility of this index, it has been widely used in the study of pseudo-allergic reactions, such as chlorogenic acid-induced cell degranulation [44] and the study of pseudo-allergic reactions to Xiangdan injection [23].

Tryptase, also known as mast cell tryptase, is currently the main MC biomarker available in medical practice. Tryptase determination is a quantitative test performed in serum or plasma for the diagnosis, stratification, and follow-up of mast cell−related conditions. The continuous secretion of monomeric a and b protease forms the baseline tryptase level. Transient, activation-induced release of tryptase is known as acute tryptase. Because mast cells are tissue-resident cells, the detection of an acute tryptase release in the bloodstream is protracted, with a delay of fifteen to twenty minutes after the onset of symptoms and a peak at approximately one hour. Constitutive release of tryptase is a marker of mast cell number and activity status, whereas transient release of mature tryptase is a marker of mast cell degranulation [45].

### Complement activation

Mast cell degranulation induced by complement activation is one of the important mechanisms in the occurrence of anaphylactic reactions, and it is also the most well-studied. Complement is a group of plasma proteins, of which there are about twenty kinds. It exists under natural conditions in the form of inactive enzymes, which decompose to produce active fragments after activation. Its activation generally involves three pathways: the classical pathway, the lectin pathway, and the bypass pathway. These three pathways are driven by a series of proteolytic reactions, which convert proenzyme into active enzyme and eventually form C3 invertase, triggering subsequent cascades that can cleave C3 into C3a and C3b, and promote the generation of more C3 molecules to amplify the effect of C3b, which is involved in the generation of C5 invertase, which can decompose C5 into C5a and C5b. The reaction is similar to C3, while C5b continues to mediate the generation of the C5B-9 complex, which is the membrane attack complex (MAC) in the host defense response. By binding to the cell membrane and embedding in the phospholipid bilayer, C5b can induce the formation of pore structures on the cell, allowing the exosmosis of intracellular substances, and finally lysis to release the active medium [46]. Studies have shown that solubilizing Tween 80, which is often used in TCMIs, causes anaphylactic reactions by activating the complement system [47], and Tween 80 is contained in Shengmai and Shenmai injection. Therefore, the occurrence of anaphylactic reactions in human serum complement system activated by Shengmai and Shenmai injection may be associated with Tween 80. Liu Xueying et al. [48] found that Shengmai and Shenmai injection could both increase the content of SC5b-9 in human serum, and the variation trend of SC5b-9 in serum caused by different blood groups was the same. Gao et al. [49] found that the mechanism of anaphylaxis with Shuanghuanglian injection was mainly related to the release of the allergenic toxin C5a through the complement system. When they administered C3a and C5a receptor inhibitors to animal models, respectively, the degree of foot cyanosis was significantly reduced in the C5a group of mice, which further suggests that inhibition of the specific receptors of allergenic toxins can reduce the degree of anaphylaxis and is expected to be a new method for the prevention and treatment of anaphylaxis.

### Coagulation system

The coagulation system specifically includes two types of exogenous and endogenous pathways, the latter one is from coagulation factor XII, which exists in the plasma in the form of proenzyme and is automatically activated by conformation reorder upon contact with negative ions on the cell surface, and this number of types of FXIIa initiate endogenous coagulation cascade reactions, and at the same time produce thrombin and fibrous eggs [50]. Fibrin promotes thrombin to activate coagulation factor XIII to accelerate the cross-linking polymerization of fibrin monomer to generate fibrin polypeptide [51], which leads to coagulation, degranulation of mast cells, histamine release, and increased vascular permeability. Thrombin also plays an essential role in allergic reactions, participating in airway inflammation and promoting fibroblast proliferation [52] by activating protein kinase receptors on the surface of mast cells. Protein kinase receptors (PKR) are also G-protein-coupled receptors, including Par1-4 types [46], in which thrombin can recognize PAR-1, 3, and 4 activation signal transduction pathways, triggering a cascade reaction that can promote mast cells to release histamine, protease, cytokines, and IL-4, IL-5, and other active mediators. This can lead to increased vascular permeability, resulting in allergic reactions such as skin mucosal edema [53], and PAR-2 is a receptor for trypsin and trypsin-related enzymes, which is closely related to mast cell degranulation reaction [54].

### Kallikase-the kalinin system

Activated FXII, the initiating serine protease in both the contact and the intrinsic coagulation systems, activates factor XI and prekallikrein, respectively. FXII-mediated bradykinin (BK) formation has been proven in the human plasma of anaphylactic patients as well as in experimental models of anaphylaxis. Moreover, the severity of anaphylaxis is correlated with the increase in plasma heparin, BK formation, and the intensity of contact system activation. FXII also activates plasminogen in the fibrinolysis system [55]. BK is one of the most effective vasodilator drugs, which can induce the release of endogenous vasodilator factors, such as NO, prostacyclin (PGI2), etc. The biological activity of bradykinin is achieved by mediating the cell-surface bradykinin receptor, which belongs to the G-protein-coupled receptor family and includes two subtypes, bradykinin receptor type 1 and bradykinin receptor type 2. Bradykinin can bind to the latter with high affinity to instantaneously activate phospholipase Cr1. In addition, this signal transduction increases vascular permeability and releases arachidonic acid, cytokines, cyclic adenosine phosphate, estrogen, glucocorticoid, and other mediators that are involved in a variety of pathological reactions such as allergies, acute pain, severe inflammation, and edema. However, the expression of the former is very low under normal physiological conditions. After activation, it is up-regulated rapidly, resulting in increased tissue damage or inflammation [56, 57].

### Others

The researchers found that TCMIs allergens can activate protein kinase C and Ca^2+^ related signaling pathways by activating G protein. When intracellular Ca^2+^ concentration increases, RBL-2H3 cells degranulated and released histamine, β-amino-hexokinase, and other biological mediators, thus causing allergenic occurrence. The study of Pangfei et al. [23] showed that the anaphylactic reactions induced by Xiangdan injection may be related to salvianolic acid B and violet oxalic acid, and may be related to the mobilization of Ca^2+^ influx.

## Research model of anaphylaxis

### Animals model of anaphylactic reactions

Current commonly used animal models for allergy research include: ICR mice, rats, beagle dogs, miniature pigs, New Zealand rabbits, cynomolgus monkeys, etc. The sensitivity and symptoms of such allergic reactions vary depending on the animal species. The strengths and weaknesses of frequently used animal models are summarized in Table 2.

**Table 2:** Advantages and disadvantages of animal models

Animals model	Advantages	Limitations	Molding method
Mice	Small size, easy access to materials, convenient feeding	Some physiological anatomy is difficult, the blood volume is small	Tail vein or hind paw administration
Rats	Medium size, high volume of blood	An individual’s genetic material is unstable	Tail-vein injection
Guinea pigs	The serum contains abundant complement and the complement is stable	Low sensitivity	Ear intravenous
Beagle dogs	Moderate body size, mild temperament, convenient and accurate administration, convenient examination and blood collection, no anesthesia	High price	Forelimb intravenous injection
Cynomolgus monkeys	Highly similar to humans in morphology, physiology and genetics	High price and ethical issues	Forelimb intravenous injection

#### Rats

Mice are small, easy to operate, convenient to obtain materials, mature technical means of genome modification, short generation cycle, which makes the experiment efficiency high, and the differences between individuals are observed in the results of parallel experiments, which is one of the commonly used animal models for the study of TCMIs pseudo-allergic reactions. Taking vascular permeability as an indicator, researchers conducted pathological examination by observing the incidence of auricle blue, staining area, Evans Blue (EB) exudation amount, edema and inflammation degree of ear lung tissue in mice, and made a comprehensive evaluation of anaphylaxis [22, 30, 59, 60]. In addition, the study of Zhang Yushi et al. [61] showed that ICR mice were a relatively ideal test strain and should be used as the preferred animal for allergy experiments of TCMIs. Next, the Kunming mice can be selected. The Balb/c and C57 mice are less sensitive and are not recommended for allergy testing of TCMIs. The allergic response to TCMIs in mice differed between the sexes, with males being more sensitive. Jiang Wenjun et al. observed the antagonistic effect of Xanthium extract on C48/80-induced pseudo-allergic reactions by measuring the swelling of mouse feet and the exudation of tissue fluid, which provided a reference for the study of pseudo-allergic reactions [62]. Lu et al. [63] developed a comprehensive metabolomics approach to study the effects of RD-induced anaphylaxis in mice using liquid chromatography-electrospray ionization-time-of-flight mass spectrometry. Changes in metabolites associated with inflammation and allergic disease were observed in the early stages of anaphylaxis, suggesting that lipid metabolism disordered, such as glycerophospholipids and steroid hormone metabolism, may be associated with RD-induced anaphylaxis.

Compared to mice, rats are larger in size and are easy to observe and record for a variety of physiological and pathological changes. Through proteomic research methods, Chen et al. extracted seven differential proteins related to pseudo-allergic reactions in BN rats, and found some candidate biomarkers related to anaphylactoid mechanisms [64]. Li et al. [65] used chlorogenic acid to study pseudo-allergic reactions in BN rats. There was no statistically significant difference in serum trypsin activity and histamine content in BN rats compared to the negative control group, and the results showed no obvious anaphylactoid reaction to chlorogenic acid. By intradermal injection of Chuanhuning injection, Li et al. [8] observed significant local edema in rats, forming large blue spots, and the results showed that Chuanhuning injection could cause significant pseudo-allergic reactions in rats, suggesting potential pseudo-allergic reactions in clinical treatment. Chen et al. [66] used the appearance of blue spots on the skin of rats as a detection index. They found that the skin allergy test method in rats is simple to perform, has a short testing period, requires a small number of drugs, exhibits high sensitivity, and yields repeatable results. However, it is important to note that the characteristics of the drug itself can sometimes lead to false positives, thus potentially affecting the test outcomes.

#### Guinea pigs

Guinea pigs are the most widely used model for allergic reactions, despite the vast differences between them and humans. Yang et al. [38] selected Guinea pigs model to study the pseudo-allergic reactions of ligustrazine phosphate injection, and determined that the contents of histamine, β-Hex and trypsin were significantly increased, indicating that ligustrazine phosphate injection had a high probability of causing pseudo-allergic reactions. Wan et al. [67] conducted a routine passive skin pseudo-allergic reactions test with Guinea pigs and found that component A of Shuanghuanglian injection could cause significant positive reactions in Guinea pigs, indicating that macromolecular substances in Shuanghuanglian injection were an essential cause of pseudo-allergic reactions caused by intravenous injection, and the mechanism involved increased vascular permeability. However, the Guinea pig’s model showed low sensitivity to anaphylaxis induced by TCM injections and is more suitable for the study of type-I allergic reactions.

#### Beagle dogs

Beagle dogs are a common animal model for anaphylaxis research, and they perform relatively well. Beagle dogs have been found to be sensitive to TCM injections, making them a sensitive animal model. Therefore, Beagle dogs can be used as candidate animal models in the study of anaphylaxis in TCMIs. He et al. [68] selected Beagle dogs to study anaphylactic reactions to Shengmai injection (new technology) components and took reaction symptoms and changes in serum histamine content after administration as the main reaction indicators, which is feasible to a certain extent. In addition, Li et al. [69] administered Xingnaojing injection intravenously to Beagle dogs, and given Xingnaojing injection in large doses, the Beagle dogs showed different degrees of anaphylaxis symptoms and ECG respiratory effects, which may be related to the excipient Tween 80. The study had shown that allergen-specific IgE/IgG was not detectable in dog serum, but there was a trend to lower total serum IgE levels (and decreased IgE: IgG ratios). Mi et al. [47] measured plasma histamine and β- Hex in beagle dogs, and the results indicate that the adverse reaction induced by vitamin K1 injection is anaphylactoid, not anaphylaxis. Vitamin K1 injection induces the release of inflammatory factors via a non-IgE-mediated immune pathway, for which the trigger may be the solubilizer. Although the price of Beagle dogs is relatively high compared to other small animal models of anaphylaxis, the sensitivity of Beagle dogs to TCMIs closely resembles that of human skin. Therefore, based on the research of other tiny animal models of anaphylaxis, it is recommended that a beagle dog can be used as one of the large animal models to evaluate the allergic reaction of TCMIs.

#### Others

In addition to the common animal models such as mice, rats, Guinea pigs and beagles, cynomolgus monkeys have also been used to study allergic reactions induced by TCM injections, but less research is currently conducted due to high prices and ethical concerns. Li et al. [70] used cynomolgus monkeys to conduct a comparative study on the allergic reactions of Shengmai injection produced by the new and old processes, and the study showed that the improved process of Shengmai injection caused the allergic reactions of cynomolgus monkeys to appear late and to a mild degree, and the sensitization to cynomolgus monkeys was reduced.

### Cell models of anaphylactic reactions

Primary mast cells or basophil granulocytes are generally used in the vitro experiments of pseudo-allergic reactions. MC is the main effector cell of pseudo-allergic reactions, which can secrete histamine and various inflammatory and immunomodulatory substances through degranulation, thereby causing physiological and pathological changes in the body [71, 72]. Most of these models take target cells as research objects to explore the release mechanism of allergenic mediators. Frequently used cell models include rat basophil leukemia-2H3 cells (RBL-2H3), P815, Ku812 cells and HUVEC cells. The advantages and limitations of commonly used cellular models are summarized in Table 3. The use of cells to assess pseudo-allergic reactions induced by TCMIs and possible mechanisms is summarized in Table 4.

**Table 3:** Advantages and disadvantages of cell models

Cell lines	Cells	Advantage	Limitations
Rat basophilic leukemia cell line	RBL-2H3	Simple operation, good stability	Not have all mast cell functions and information
Mouse mastocytoma cell lines	P815		
Human peripheral blood basophilic leukemia cell lines	Ku812	Able to be stably cultured	Slightly less sensitive, cell degranulation condition should not be observed
Human umbilical vein endothelial cell lines	HUVEC	Easy access, no ethical controversy	The growth rate is slow and it takes a long time to reach the logarithmic growth period

**Table 4:** Cellular evaluation of herbal injections and possible mechanisms

Cell	TCMI	Mechanism	References
RBL-2H3	Qingkailing injection	Based on PI3K-Rac1 signaling pathways	Li Q, [77]
	Sodium hesperidin for injection (SAI)	\	Wang D. [82]
	Thromboxane for Injection (lyophilized) and Phlegm Fever Clear Injection	Direct action and bypass pathway via activation of the complement system	Song Y. [83]
	Yiqi Fumai for Injection (Lyophilized)	\	Fan S, [84]
	Shuanghuanglian injection	\	Wang F, [85]
	Shegan antiviral injection	Associated with increased intracellular Ca2 + concentration	Zhang RR, [78]
	Shenmai injection	May be related to the solvent Tween-80	Xiong K, [86]
	Mao Dongqing injection	\	Fan NQ, [87]
P815 cell	Xiangdan injection	May be related to the mobilization of Ca^2+^ inward flow	Pang F, [23]
HUVECs cell	Shuxuening injection	hyperactivation of the mTOR signaling pathway	Wang L, [8]
RBL-2H3 and P815 cell	Vitamin K1 injection	\	Xu M, [88]

#### RBL-2H3 cells

RBL-2H3 cells possess immortal properties, a simple culture method, rapid growth, and avoid the tedious step of gradient separation of basophilic granulocytes from blood. Therefore, RBL-2H3 cells are a good cell model for the establishment of type I allergy and anaphylaxis in vitro [73]. Zhu et al. [74] conducted a feasibility study on the allergic reaction model of Yinzhihuang injection using the RBL-2H3 cell model, and the results showed that RBL-2H3 cells could be used in the in vitro model of allergic reaction detection, and the histamine release amount of cell supernatant and Ca^2+^ fluorescence intensity could be used as evaluation indexes. Fu et al. [75] used the RBL-2H3 cell model to screen the main allergenic components of Honghua injection, and the research results suggested that the sensitization of Honghua injection was related to the allergenic mechanism, and the fat-soluble components were closely related to the occurrence of allergic reactions. Han et al. [76] established a RBL-2H3 and allergic disease 2 (LAD2) dual-mixing/CMC laboratory and used IT in combination with HPLC-ESI-ITO-ToF-MS system to identify potential allergenic components in Haqing injection. Li et al. [77] evaluated that the pseudo-allergic reaction induced by Qingkailing injection was based on the PI3K-Rac1 signaling pathway by using RBL-2H3 cells. Zhang et al. [78] studied the pseudo-allergic reactions induced by Shegan antiviral injection at animal and cellular levels and found that Shegan antiviral injection has the risk of triggering pseudo-allergic reactions, and the mechanism may be related to the increase of intracellular Ca^2+^ concentration.

#### P815 cells

Mouse mastocytoma cells (P815) are stable and cultured mast cell lines. Due to their special properties of mast cells, they are gradually used as models for in vitro studies of allergic reactions. Compared to basophils, MCs release a greater number of biological mediators in allergic and pseudo-allergic reactions, and more of them are found in the human body than basophil cells. Therefore, taking mast cell lines as research objects can improve the relevance of model prediction results to the clinic. Pang et al. [23] used the P815 model to screen out possible allergenic components in Xiangdan injection and elucidate their mechanism of action. Liu et al. [79] utilized P815 cells as the allergy model in vitro, and the results showed that chlorogenic acid and caffeic acid contained in Xuebijing injection could significantly cause the degranulation of P815 cells and the release of histamine and aminoglycoside, which could provide a preliminary reference for the evaluation of hypoallergenic effects of Xuebijing injection to a certain extent.

#### Ku812 cells

Human peripheral blood basophilic leukemia cells (Ku812) are a kind of human immune cell that can be statically cultured in vitro, and can be used as a preliminary detection method for drug allergic reactions. The method is simple, rapid and sensitive, and is suitable for the preliminary screening of batch samples. However, Ku812 is a suspended cell and degranulation conditions are not easily observable. Yang et al. [80] compared the effect of different concentrations of Tween-80 solution on the degranulation of RBL-2H3, P815 and Ku812 cells, and the results showed that degranulation of the three cell models was significant with the increase of Tween-80 concentration, but compared with Ku812 and P815 cells, RBL-2H3 cells are more suitable for mast cell degranulation detection model in vitro, suggesting that Ku812 cell line is slightly less sensitive in the preliminary screening of allergenic ability and has few practical applications.

#### Human umbilical vein endothelial cells

Human umbilical vein endothelial cells (HUVECs) are derived from neonatal umbilical cord tissue, which is easy to obtain, without ethical controversy, and easy to extract sufficient cells, and is also suitable for the study of pseudo-allergic reactions induced by Chinese medicine injections. Han et al. [81] established a method of incubating HUVEC monolayer with Shuanghuanglian injection, using endothelial permeability and cytoskeleton changes as observation indexes, and combined with western blot analysis, and found that Shuanghuanglian injection could induce anaphylactoid reactions.

## Conclusion and outlook

TCMIs play a crucial role in the modernization of TCM. However, its safety has always been the focus of public attention, which has become a bottleneck hindering the development of new drugs in TCMIs. First, in view of the high incidence of pseudo-allergic reactions induced by traditional Chinese medicine injection, we summarize the causes of pseudo-allergic reactions, including the differences in traditional Chinese medicinal materials, complex components, the production process, dosage, and injection speed of TCMIs. Second, the composition of TCMIs is complex, and its specific allergenic components are not clear. This review summarizes the allergenic components in traditional Chinese medicine injections, including chlorogenic acid, Tween-80, tannins, > 10 kDa molecules (proteins), baicalein, Arctiin, etc. Third, there is more than one mechanism by which TCM-induced pseudo-allergic reactions occur, and the same TCM injection may have two or more sensitizing mechanisms. This review summarizes the research status on the mechanisms of anaphylaxis and biomarkers for evaluating anaphylaxis. Currently, the biomarkers commonly used to assess pseudo-allergic reactions are histamine, β-Hex, tryptase, and specific IgE, and 1–3 biomarkers are usually selected for evaluation. Finally, there is no uniform and clear regulation of the evaluation method of pseudo-allergic reactions currently, and researchers mainly evaluate based on cell models and animal models. In this review, we summarize the evaluation methods for pseudo-allergic reactions, analyze the strengths and weaknesses, and select appropriate models based on the experimental conditions and the experimental design.

Exploring the mechanism of pseudo-allergic reactions, finding more reliable biological diagnostic indicators, strengthening the application of new technologies in research methods, and establishing recognized detection methods and models for pseudo-allergic reactions are the next research priorities. The mechanism of pseudo-allergic reactions has made some progress, and its upstream and downstream pathways can be explored more deeply by network pharmacology, metabolomics and proteomics [89]. At present, there are still some limitations in the study of sensitizing components of TCMIs with complex components. The development of rapid, sensitive and high-throughput screening methods for sensitizing components, and the improvement and refinement of quality standards are the major needs currently faced by China's TCMI industry. Based on the previous research, with the sensitizing targets IgER, H1R and MrgprX2 as the research core, combined with high-performance liquid chromatography-mass spectrometry, high-throughput, high-sensitivity micro dialysis mass spectrometry signal attenuation technology, the potential allergenic components of TCMIs can be quickly identified in vivo and can be identified at the cellular and animal levels, providing key support for their quality control and safety evaluation, and also providing new technologies and methods for the research of allergenic components in TCMIs. Meanwhile, the screening efficiency of allergenic components in TCMIs can be improved by the unique advantages of cell membrane chromatography (CMC), microarray technology and computer-assisted virtual screening technology in the screening of complex components of traditional Chinese medicine [90–92] This has important implications for the safety assessment and clinical rational use of TCMIs.

## Data Availability

Not applicable.

## Abbreviations

TCMIs: Traditional Chinese medicine injections
TCM: Traditional Chinese medicine
EB: Evans Blue
IgE: Immunoglobulin E
ADR: Adverse drug reaction
MCs: Mast cells
MrgprX2: MAS-associated G-protein-coupled receptor X2
β-Hex: β Hexosaminidase
MAC: Membrane attack complex
PKR: Protein kinase receptors
BK: Bradykinin
RBL-2H3: Rat basophil leukemia-2H3
PGI2: Prostacyclin
HUVECs: Human umbilical vein endothelial cells
CMC: Cell membrane chromatography
